# Wear Resistance and Biocompatibility of Co-Cr Dental Alloys Fabricated with CAST and SLM Techniques

**DOI:** 10.3390/ma15093263

**Published:** 2022-05-02

**Authors:** Wenqi Fu, Shuang Liu, Jun Jiao, Zhiwen Xie, Xinfang Huang, Yun Lu, Huiying Liu, Shuhai Hu, Enjun Zuo, Ni Kou, Guowu Ma

**Affiliations:** 1 Department of Oral Prosthodontics, School of Stomatology, Dalian Medical University, Lvshun South Road, Dalian 116044, China; fwq0369@163.com (W.F.); hdllsphsss@163.com (S.L.); junjunjiaojun@126.com (J.J.); lyyida2009@126.com (Y.L.); lhy04512000@163.com (H.L.); shuhaihu4141@aliyun.com (S.H.); zuoej@163.com (E.Z.); 2 Academician Laboratory of Immune and Oral Development & Regeneration, Dalian Medical University, Lvshun South Road, Dalian 116044, China; 3 School of Mechanical Engineering and Automation, University of Science and Technology Liaoning, Anshan 114051, China; xzwustl@126.com (Z.X.); xfgg527@163.com (X.H.)

**Keywords:** dental restoration wear, biocompatible, casting technics, cobalt–chromium alloys, selective laser melting

## Abstract

Cobalt–chromium (Co-Cr) alloys have been widely used as dental-restoration materials for many years. This study sought to investigate whether selective laser melting (SLM) is a more appropriate process than traditional casting (CAST) for fabricating dental Co-Cr alloys. Metallurgical microscopy, X-ray photoelectron spectroscopy (XPS), Vickers hardness and nanoindentation tests, and friction and wear tests were used to evaluate the microstructure, surface compositions, mechanical properties, and wear resistance, respectively. Additionally, the biocompatibilities and cell adhesion of the alloys were evaluated with L-929 fibroblasts via CCK-8 assay, Live/Dead staining, flow cytometric analysis, scanning electron microscopy (SEM) observation and real-time PCR (RT-PCR) assay. The XPS results showed that the two alloys were all mainly comprised of Co, Cr, and O. The hardness in the CAST group equaled 7.15 ± 0.48 GPa, while in the SLM group, it equaled 9.06 ± 0.49 GPa. The friction coefficient of SLM alloys remained at approximately 0.46, but the CAST specimens fluctuated significantly. SLM alloys exhibited shallower wear scars and less wear debris compared with CAST alloys, simultaneously. Additionally, there were higher survival and expression of cell-adhesion-related genes on SLM alloys of L-929 cells, which meant that the deleterious effect on L-929 cells was significantly reduced compared with that for the CAST alloys. Overall, the wear resistances and biocompatibilities of the Co-Cr dental alloys were dramatically affected by the fabrication technique. The SLM technique is advantageous over the CAST technique for fabricating Co-Cr dental alloys.

## 1. Introduction

Co-Cr alloys have been increasingly used for dental restorations owing to their excellent mechanical properties, corrosion resistance, and metal–ceramic combination [1]. However, the flaws, porosity, and costs arising from the complex steps of the traditional lost-wax casting technique seriously limit its applicability [2,3,4,5]. Simplification of the procedures can mitigate these errors, along with cost of improving the restoration quality. Computer-aided design/computer-aided manufacturing (CAD/CAM) provides advanced routes for alloy processing as an alternative to the CAST [6]. The impression-taking procedure can be avoided via the development of direct oral-scanning devices, improving the fabrication-time efficiency and the precision of the dental restorations. Depending on the processing method, it can be divided into subtractive and additive processes. The subtractive method is widely applied for dental restorations, but it has a limited potential for obtaining complex shapes and results in a substantial waste of material [7]. As for the additive manufacturing, it is a kind of powder-bed fusion technology, including selective laser sintering (SLS), selective laser melting (SLM), and electron beam melting (EBM) [8]. Because there are better mechanical properties and higher accuracy of the restorations prepared by SLM technology compared with those provided by SLS technology and the EBM method, SLM technology is the most extensively used, such as in applications for implants, porcelain fused to metal (PFM), and removable partial-denture frameworks [9]. It produces restorations directly from three-dimensional (3D) CAD models by fusing fine layers of metal powder using a high-power focused laser beam [10], which has the advantage of reducing the human and technical errors in the manufacturing process and leads to a higher restoration quality [11].

Co-Cr dental alloys fabricated via the SLM technique exhibit different microstructures and mechanical properties from similar alloys produced via the CAST technique [10,12,13]. Limited information is available regarding the wear resistances, biocompatibilities and cell early adhesion of Co-Cr dental alloys prepared via CAST and SLM techniques. Metal restorations produce small wear debris or metal particles when repeated friction with the counter teeth is used to perform mastication. These debris and particles can react with body fluids, causing local tissue inflammation [14]. A growing number of studies have focused on improving the wear resistance via surface modification and different manufacturing techniques [15,16,17,18,19,20]. Surface-modification technology can retain the excellent mechanical properties of the substrates and improve the wear resistance of materials [16]. However, these methods require the bond-strength problem to be solved. Different manufacturing techniques can be used to alter the mechanical properties of alloys by changing the material microstructure, affecting the wear resistance [13]. The biocompatibility of the alloy is closely related to its composition and structure. Co-Cr alloys release metal elements such as Co, Cr, and Mo, which may cause inflammatory reactions, discoloration, and hyperplasia in the adjacent tissue [21,22]. The extent of metal release depends on the fabrication procedures [23]. In addition, there have been many studies on the mechanical properties and biocompatibility of dental materials [24]. The present study further focuses on the effect of different processing techniques on cell adhesion. Surface topography influences the cell shape, adhesion and spreading, which are different depend on the manufacturing techniques. Restorations remain in close contact with the gingival tissue; as the foreign body, the formation of early cell adhesion is beneficial to recover the tissue surrounding the restorations. VEGF and Col-I are two important factors to regulate the cell initial adhesion [25]. Thus, it is necessary to evaluate the biocompatibilities and cell-adhesion behavior of alloys fabricated using different techniques.

To address this issue, the addition of microstructures, chemical compositions, mechanical properties, and wear resistances of alloys fabricated were investigated via two techniques, as well as cell proliferation, cytotoxicity, cell apoptosis, cell morphology, and adhesion-related gene expressions of L-929 cells, to evaluate their feasibility for dental applications. Based on their excellent wear resistance and biocompatibility, Co-Cr dental alloys prepared via the SLM technique are comparable to—or even better than—those prepared via the CAST technique. Thus, the first null hypothesis of this study is that there are no differences in the mechanical properties relative to the manufacturing techniques. The second null hypothesis is that there are no differences in the biocompatibility, such as the cell proliferation, cell apoptosis, and cell adhesion of L-929 cells, between the CAST and SLM alloys.

## 2. Materials and Methods

### 2.1. Specimens Preparation

**Figure 1:**
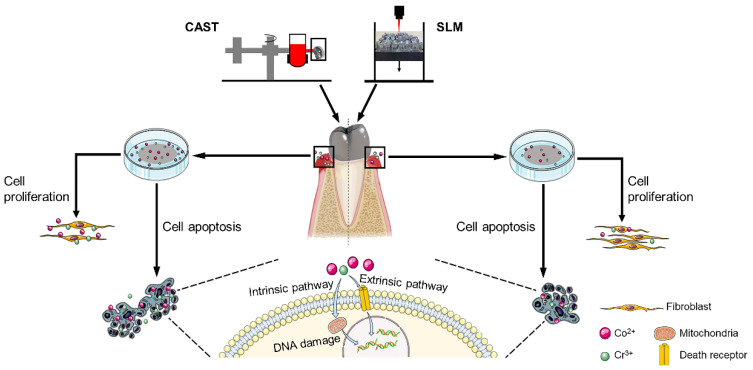
Schematic illustration of the experimental procedure.

Commercially available Co-Cr disks (Ruijia, Beijing, China) with a diameter of 20 mm and a thickness of 1 mm were fabricated with the CAST and SLM techniques, serving as CAST and SLM group, respectively. The specimens were sanded with SiC abrasive papers progressively up to 2000 grit and then polished to a mirror finish. Subsequently, they were ultrasonically cleaned in acetone, absolute alcohol, and deionized water for 15 min to remove surface contaminants and dried. The Schematic illustration of the experimental procedure was shown in Figure 1.

### 2.2. Alloy Characterization

#### 2.2.1. Metallurgical Microscopy

Metallurgical microscopy (Carl Zeiss Image AIM, Jena, Germany) was utilized to observe the microstructures of the specimens. Prior to examination, each group of specimens was etched for 60 s using hydrogen nitrate/hydrochloric acid (1:3, v/v) at room temperature. Micrographs were taken at 100× magnification.

#### 2.2.2. X-ray Photoelectron Spectroscopy (XPS)

To determine the surface chemical compositions and states, XPS (K-Alpha, Thermo, Waltham, MA, USA) was performed using monochromatic Al Kα X-rays with an energy of 1487 eV. The core-level signals were obtained at a photoelectron takeoff angle of 45°. The energy spectrum was corrected using C1s (284.8 eV). The wide-scan spectra were recorded in steps of 1 eV and a pass energy of 150 eV, while high-resolution spectra with steps of 0.1 eV and a pass energy of 20 eV. The elemental compositions of the alloys were determined with reference to standard spectra obtained from the XPS International Inc. website. The XPSPEAK 4.1 software was employed to analyze the data using the Gauss–Newton fitting method.

#### 2.2.3. Mechanical-Property Tests

The microhardness of the alloys was measured using a Vickers hardness tester (Q10M, QNESS, Colling, Austria). The load in the test was 200 g, with a dwell time of 10 s. Each specimen was tested at three points, and the results were averaged.

The nanohardness and elastic modulus were determined via nanoindentation tests (G200, KLA-Tencor, Milpitas, CA, USA), and the ratio of the hardness to the elastic modulus (H/E) was calculated. The indentation depth was 2000 nm, and the results of at least 10 indentations were averaged to improve the reliability of the results.

#### 2.2.4. Friction and Wear Tests

A ball-on-disc tribometer (MS-T3000, Lanzhou, China) was used to investigate the wear properties of the specimens under dry conditions. Each specimen was tested using a silicon nitride ceramic ball (Si_3_N_4_, φ6 mm), with the following parameters: a load of 5 N, a sliding speed of 200 rpm, and a test duration of 30 min. The friction coefficients were recorded, and the wear scars were observed using an ultra-depth 3D microscope (VHX-500F, Keyence, Osaka, Japan). Three duplicate specimens were tested for each group.

### 2.3. In Vitro Biocompatibility Evaluation with L-929 Cells

#### 2.3.1. Metal-Extract Preparation

Metal extracts were prepared by immersing the specimens in a culture medium for 72 h at a ratio of 3 cm^2^/mL according to the ISO 10993 guidelines [26].

#### 2.3.2. Cell Culture

Mouse fibroblasts L-929 were cultivated in a MEM medium (BI, Israel) with 1% antibiotics (Gibco, Thermo Fisher, Waltham, MA, USA) and 10% fetal bovine serum (Gibco, Thermo Fisher, Waltham, MA, USA) at 37 °C in a 5% humidified CO_2_ atmosphere. Cells seeded on the normal medium were considered as the control group, and cells cultivated in a medium with CAST and SLM alloy extracts served as the experimental groups, which were denoted as “CAST” and “SLM”, respectively.

#### 2.3.3. CCK-8 Assay

The proliferation of L-929 cells was evaluated via a CCK-8 assay. Cells were seeded on the surface of samples at 5 × 10^4^/mL and cultured for 1, 3, and 5 d. After incubation for the corresponding time, the samples were moved into a new 12-well plate and incubated in 10% CCK-8 (Abbkine, Redlands, CA, USA) containing medium for 2 h at 37 °C. The optical density (OD) was measured at a wavelength of 450 nm using a microplate reader (Thermo, Waltham, MA, USA). Both cell assays were performed at least in triplicate.

#### 2.3.4. Live/Dead Staining

Cytotoxicity was determined by staining L-929 cells using a Live/Dead cell double-staining Kit (Abbkine, Redlands, CA, USA). After being cultured for 24 and 48 h, the cells were stained with the staining solution for 15 min in the dark. Live cells were stained in green and dead cells were red. Then, the cells were observed with a fluorescence microscope.

#### 2.3.5. Flow Cytometric Analysis

The cell apoptosis was measured via Annexin V-FITC/PI staining (Invitrogen, Waltham, MA, USA) and Flow cytometric analysis. Cells were cultured in normal medium or alloy extracts for 48 h and then incubated with 5 μL of Annexin V-FITC and 5 μL of propidium iodide (PI) at room temperature and kept in a dark environment for 15 min. The cells were then analyzed via flow cytometry (Guava, Luminex Corporation, Austin, TX, USA). The FlowJo7.6 software was used to analyze the flow cytometric data, which were displayed in a dot plot of Annexin V-FITC/PI staining.

#### 2.3.6. SEM Observation

The morphology of L-929 cells adhered on samples was observed by FE-SEM (Supra 55, Zeiss, Germany). Cells were seeded on samples at 5 × 10^4^/mL and cultured for 3 and 5 d. The samples were dehydrated through gradient ethanol solutions (30%, 50%, 70%, 80%, 90% and 100%) after fixed in 4% Paraformaldehyde, and air-dried at room temperature. The cells were gold-sputtered and then observed via FE-SEM.

#### 2.3.7. RT-PCR Assay

To further confirm the influence of different alloys to induce cytokine production, RT-PCR was performed to analyze the temporal expression of vascular endothelial growth factor (VEGF) and collagen type I (COL-I ) of L-929 cells on different surfaces. The samples were processed at 3 and 5 d. Briefly, total RNA of cells cultured on the samples was extracted by using Trizol reagent (Invitrogen, Waltham, MA, Australia) and the gene expressions of VEGF and Col-I were detected by a real-time PCR machine (ThermoFisher, Waltham, MA, USA). The fold change in gene expression was calculated as 2^-ΔΔCT^. The primer sequences of each gene were listed in Table 1 with GAPDH as a housekeeping gene.

### 2.4. Statistical Analysis

All data were presented as means ± SD, the data were analyzed using SPASS 25.0 software. One-way analysis of variance (ANOVA) was used to compare differences between the specimens, T test was used to compare differences between two kinds of specimens, and differences were considered statistically significant at *p* values *<* 0.05.

## 3. Results

### 3.1. Metallurgical Microscopy

**Figure 2:**
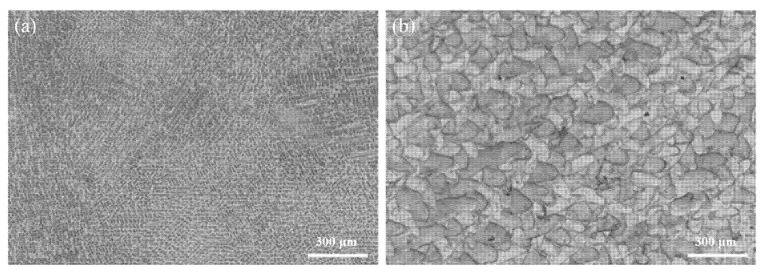
Metallurgical-microscopy images of the Co-Cr alloys produced via (**a**) CAST and (**b**) SLM.

The different microstructures of the CAST and SLM alloys are shown in Figure 2. The CAST specimens exhibit typical dendritic microstructures consisting of dendrites (light areas) and interdendritic regions (dark areas) (Figure 2a). In contrast, the SLM specimens do not have dendritic structural patterns; rather, they exhibit a scale-like structure that is homogeneous and regular (Figure 2b).

### 3.2. XPS Analysis

**Figure 3:**
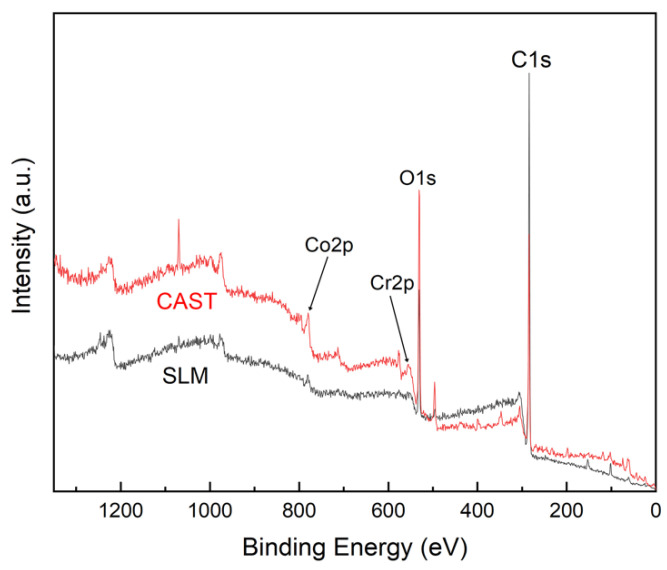
XPS spectra of the Co-Cr alloys.

**Figure 4:**
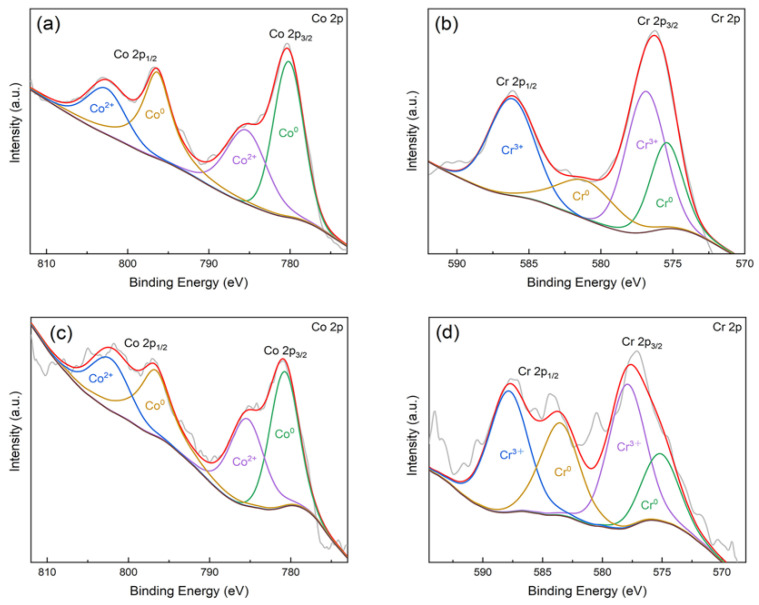
Fitting results of Co 2p and Cr 2p high-resolution spectra of the Co-Cr alloys: (**a**) Co 2p (CAST); (**b**) Cr 2p (CAST); (**c**) Co 2p (SLM); (**d**) Cr 2p (SLM).

As shown in Figure 3, the main XPS peaks for the surfaces of the CAST and SLM specimens correspond to Co 2p, Cr 2p, and O 1s, respectively. High-resolution profiles of the Co 2p and Cr 2p peaks are presented in Figure 4a–d. The Co 2p and Cr 2p peaks indicate the presence of both oxide and metal states, respectively, in all the specimens. The Co 2p peaks are attributed to Co^0^ and Co^2+^, and the Cr 2p peaks are attributed to Cr^0^ and Cr^3+^. The higher-binding energy Co 2p peak indicates the formation of Co^0^ 2p_3/2_ at 780 eV and Co^0^ 2p_1/2_ at 796 eV, and the Cr 2p peak indicates the formation of Cr^3+^ 2p_3/2_ at 577 eV and Cr^3+^ 2p_1/2_ as 587 eV.

### 3.3. Mechanical Properties

**Figure 5:**
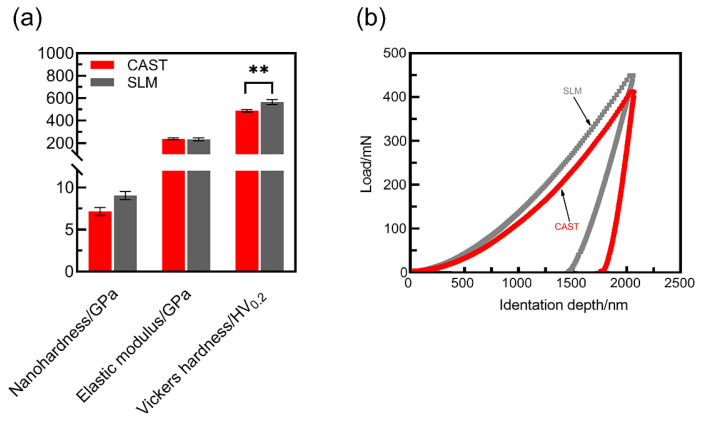
Mechanical properties of the Co-Cr alloys: (**a**) Nanohardness, elastic modulus and Vickers hardness; (**b**) Load–displacement curves (** *p* < 0.01).

The nanohardness, elastic-modulus and Vickers hardness values of the SLM and CAST specimens are plotted in Figure 5a. Compared with the CAST alloys, for the SLM alloys, the surface nanohardness is higher (CAST: 7.15 ± 0.48 GPa; SLM: 9.06 ± 0.49 GPa), the surface elastic modulus is slightly lower (CAST: 231.91 ± 12.62 GPa; SLM: 238.13 ± 8.78 GPa). Additionally, the Vickers hardness of the SLM specimens is 565.33 HV0.2, which is significantly higher than that of the CAST specimens, i.e., 487.67 HV0.2 (*p* < 0.05). The H/E ratio for the SLM group (0.039) is higher than that for the CAST group (0.031). The high H/E ratio indicates that the material has good wear resistance. The load–displacement curves of the specimens are shown in Figure 5b. Continuous curves are observed throughout the loading and unloading stages, indicating that no abrupt cracking occurred during the process. At the same load, the indentation depth is smaller and the elastic recovery is larger for the SLM alloys than for the CAST alloys, implying a higher resistance to plastic deformation due to the SLM-manufacturing technique.

### 3.4. Wear Resistance

**Figure 6:**
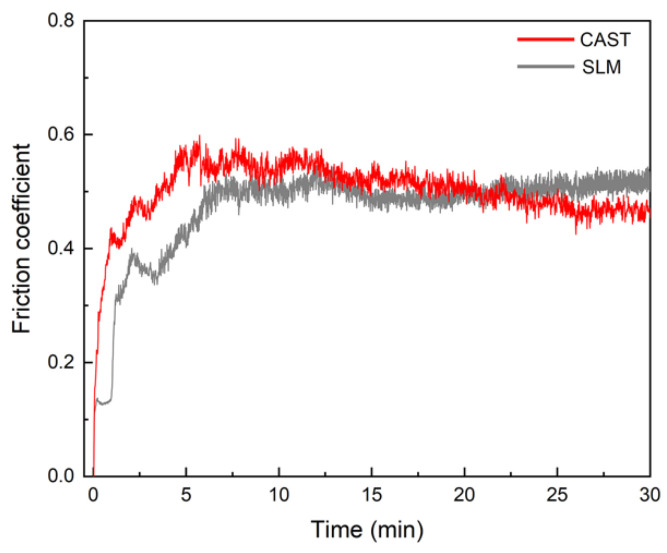
Friction coefficient curves.

The SLM alloys exhibit a higher wear resistance than the CAST alloys. The friction coefficients of the specimens were measured, as shown in Figure 6. For the CAST specimens, the friction coefficient fluctuates significantly, and the average value is 0.50. In contrast, for the SLM specimens, the friction coefficient remains at approximately 0.46.

**Figure 7:**
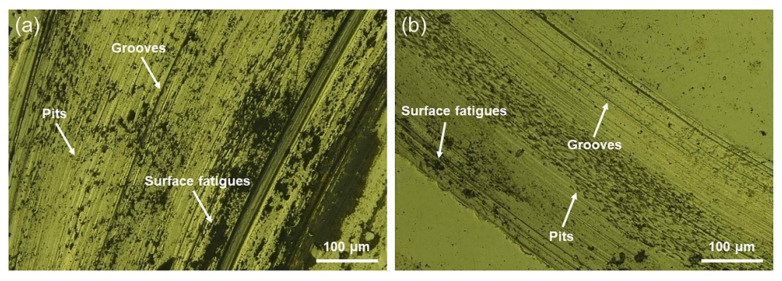
Metallurgical-microscopy images of the wear scars: (**a**) CAST and (**b**) SLM.

The wear scars were examined to identify the governing wear mechanisms for the CAST and SLM alloys, as shown in Figure 7. Plastic deformation, parallel grooves, and surface fatigue in the sliding direction are observed on the worn surfaces of all the tested specimens, implying that abrasive and fatigue wear mechanisms occurred under the test conditions. The surface of the CAST specimens exhibits large partial exfoliation and metal wear debris. The grooves on the worn surface of SLM specimens formed by the plowing action and microcutting are significantly shallower than the grooves in the CAST specimens, and the degree of plastic deformation is lower for the SLM specimens.

### 3.5. Cell Proliferation and Cytotoxicity

**Figure 8:**
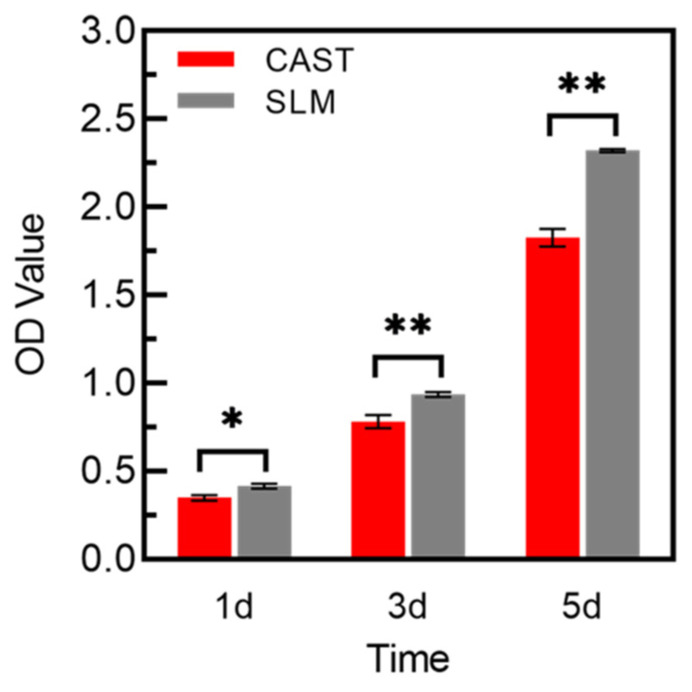
CCK-8 assay of L-929 cells cultured on the samples for 1, 3, and 5 d (* *p* < 0.05, ** *p* < 0.01).

**Figure 9:**
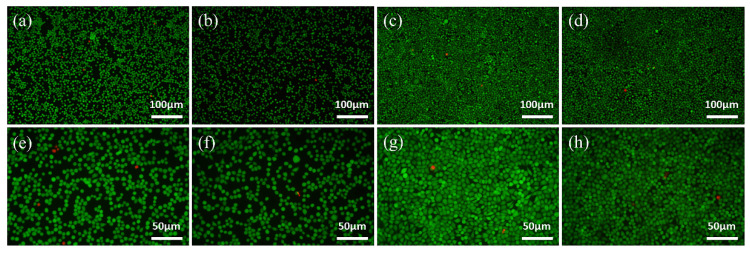
Fluorescence images of L-929 cells cultured on the samples for 24 h (**a**,**b**,**e**,**f**) and 48 h (**c**,**d**,**g**,**h**) via Live/Dead staining, indicating live cells (green) and dead ones (red): (**a**–**d**) CAST and (**e**–**h**) SLM.

As shown in Figure 8, the results of the CCK-8 tests reflect the proliferation of L-929 cells, which present a constant increase in the OD value from 1 to 3 d for all of the samples. OD values of SLM group are significantly higher than that of CAST group at all culturing time points. Green fluorescent points representing viable cells are uniformly distributed and the number of living cells increases from 24 h to 48 h in all samples, which is displayed in Figure 9. After 24 h and 48 h of culturing, a few red points exist, which indicates that some cells were dead. However, there is no significant difference between the two groups.

### 3.6. Cells Apoptosis

**Figure 10:**
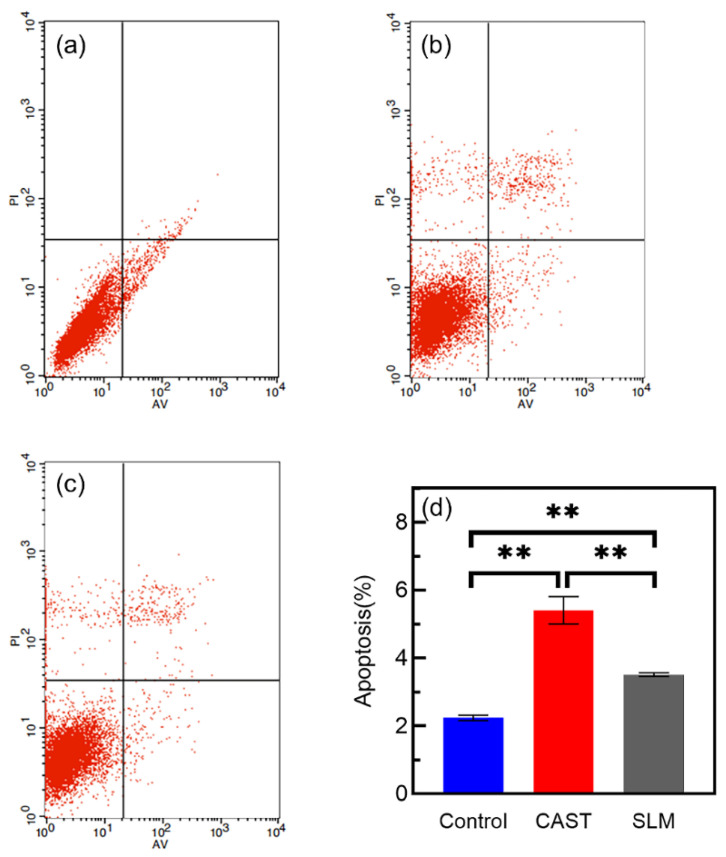
Annexin Ⅴ-FITC/PI staining and flow-cytometry analysis of L-929 cells cultured in different extracts for 48 h: (**a**) control; (**b**) CAST; (**c**) SLM; (**d**) cell apoptosis rates (** *p* < 0.01).

The apoptotic behavior of L-929 cells after culturing on each extract for 48 h is determined via Annexin V-FITC/PI staining and flow cytometry. As shown in Figure 10a–c, all the groups mainly consist of living cells, and larger numbers of apoptotic cells are observed in the CAST. The apoptosis rate is significantly lower for the SLM (3.513% ± 0.055, *p* < 0.05) than for the CAST (5.407% ± 0.401), as shown in Figure 10d. A reduced deleterious effect on the cells is observed for the SLM, indicating that the SLM alloys have better biocompatibility than the CAST alloys.

### 3.7. Cell Adhesion and Morphology

**Figure 11:**
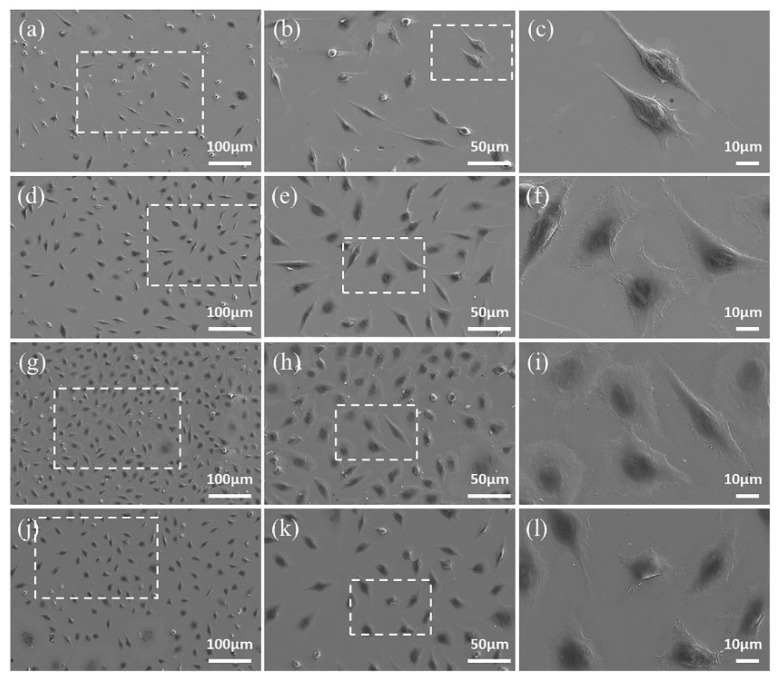
FE-SEM images of L-929 cells after culturing on CAST samples for 3 d (**a**–**c**) and 5 d (**g**–**i**), and on SLM samples for 3 d (**d**–**f**) and 5 d (**j**–**l**). The images obtained at higher magnification are taken from the areas enclosed by a square in the images taken at lower magnification.

The SEM morphology of L-929 cells cultured on samples for 3 and 5 d are shown in Figure 11. Cells cultured on the SLM samples show flat morphologies and extended lamellipodia, while tend to be spindle-like in shape and poorly spread on the CAST samples at 3 d. The number of cells increases with longer time in culture, which is consistent with the results of CCK-8 assay and live/dead staining. In addition, the cells on both samples appear elongated in shape and more extensively spread after 5 d of incubation.

### 3.8. The Gene Expression of VEGF and COL-I 

**Figure 12:**
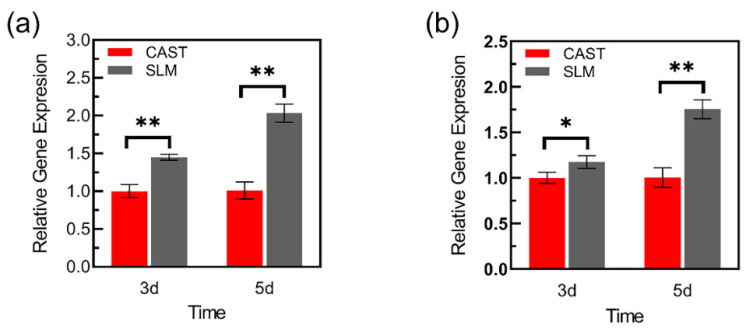
Adhesion-related gene expressions of L-929 cells cultured on the samples: (**a**) VEGF, (**b**) Col-I (* *p* < 0.05, ** *p* < 0.01).

Figure 12 displays VEGF and COL-I expressions at 3 and 5d. At both time points, the L-929 cells on SLM samples show significantly higher mRNA expression of VEGF and a similar trend is observed on the expression of COL-I. Additionally, compared with the results of 3 d, there is more pronounced difference in gene expressions at 5 d.

## 4. Discussion

The oral environment is a complex electrolyte environment with variable pH values, in which metal restorations release metal ions [27]. These ions might induce cell apoptosis via intrinsic and extrinsic pathways [28]. Dental alloys require excellent wear resistance and biocompatibility, which can extend the service time of the restorations. Co-Cr alloys have been widely used in the fabrication of frameworks for removable partial dentures, PFM, and metal crowns. Because restorations remain in close contact with the gingival tissue over long periods of time [29], in addition to studying the microstructure, components, and mechanical and tribological properties of alloys fabricated via CAST and SLM techniques, L-929 cells are used to evaluate the biocompatibility in the present study. The results of this study indicated that the wear resistance of the SLM alloys was superior to that of the CAST alloys, and the deleterious effect on L-929 cells was significantly reduced compared with that for the CAST alloys.

The microstructure, mechanical properties, and wear resistance are closely related. Manufacturing techniques affect the microstructure of Co-Cr alloys. SLM specimens have a homogeneous microstructure and low porosity owing to local melting and rapid solidification of metallic powder with high cooling rates (103–108 K/s), while CAST alloys have a characteristic dendritic microstructure, probably because of the moderate cooling rates (20–100 K/s) [5,30,31]. SLM alloy structures free of internal porosity and with a more homogeneous microstructure have superior mechanical properties to CAST alloys [32], and the wear resistance is considered to depend on the mechanical properties [33,34]. The wear debris difficultly detach from the surface and the furrows formed on the alloy surface are shallow for the alloys with high hardness. In this study, the hardness of the SLM specimens is considerably higher than that of the cast specimens. This result is consistent with previous studies [35]. Additionally, because of their high H/E ratios, the SLM specimens have a higher resistance to plastic deformation, thereby avoiding severe mechanical abrasion and friction shear during the friction process [36]. In contrast, the CAST specimens have low H/E values, and the subsurface layer of the friction contact interface is prone to strain accumulation and stress concentration, which result in severe grooves and surface fatigue.

Good wear resistance is one of the most important properties for alloys to be used as dental alloys [37]. As shown in the results of the friction and wear tests, the SLM alloys have better wear resistance than the CAST alloys. The variations in the measured friction coefficients of the samples are divided into three main periods, as indicated by the friction coefficient curves. In the first period, the friction coefficient increased immediately upon sliding owing to the start of the test. The second period of sliding was suggested to be the running-in regime, and the duration of the running stage was approximately 10 min. This stage corresponded to adventitious phenomena, which arose from the surface oxide layer formed spontaneously as a result of the exposure of the alloys to ambient O_2_ after polishing [38,39]. However, continuous wear removed the surface layer, reducing the friction coefficient. Once these surface layers were removed, the curve entered a stable wear stage, and the friction coefficient reached a steady-state value of 0.45–0.55, which is typical for metallic friction [38]. Each increase in the curves caused by the high stress resulted in the detachment of wear debris. As the debris detached, the friction coefficient decreased. Because of the severe wear that occurred on the CAST alloys, this phenomenon occurred several times, leading to wavy curves. This was confirmed by the deeper grooves and a larger degree of desquamation on the CAST alloy surface compared with those on the SLM alloy surface, as shown in the observation of the worn surfaces.

Dental materials are supposed to regulate the local oral microenvironment via contact with surrounding tissues, which are used as permanent prosthetic materials [40,41]. Of these, Co-Cr alloys release metal elements into the gingival tissue, such as Co, Cr, and Mo [21,22]. Thus, it is absolutely necessary to evaluate biocompatibility of Co-Cr alloys. Based on the results of cell proliferation and cytotoxicity, the number of cells increased with longer time in culture, indicating that metal extracts of the specimens had no obvious toxic influence on the L-929 cells. In addition, cells on the SLM specimens exhibited significantly higher viability than those on the CAST samples at all culturing time points. Flow cytometry was used to further confirm the effects of the Co-Cr alloys in fibroblasts. The cell apoptosis rate of SLM group was significantly lower than that of CAST group, indicating a reduced deleterious effect on cells, which was consistent with the results of CCK-8 assay and Live/Dead staining. These results are in agreement with recent studies indicating that the SLM technique provides Co-Cr alloys with fewer metal ions released in the medium than CAST alloys [24]. Moreover, besides the influence of releasing metal ions, previous studies indicated that the surface topography can also affect the cellular response, such as survival, attachment, and growth [42]. Dental materials might have a direct effect on surrounding soft tissues because of contact with the marginal gingiva [40]. As mentioned above, there is a more homogeneous microstructure in the surface of SLM specimens compared with CAST specimens, which might be beneficial for cell adhesion and spread. The observation of SEM shows that cells on SLM samples formed flat morphology and extended lamellipodia at the early stage, while those on CAST still remained spindle-like in shape and poorly spread. All the results indicated that the SLM alloys facilitated the initial L-929 cells adherence and these were in agreement with previous findings, which indicated that SLM alloys increased cell-adhesion ability. It is known that fibroblasts adhere to material surfaces by focal adhesion. VEGF is an important regulatory factor to promote the formation of focal adhesion, which can activate focal adhesion kinase (FAK) and assembly of focal adhesions via inducing VEGFR2 [43,44,45]. Additionally, as a major component of extracellular matrix (ECM) [46], Col-I also plays a key role in the fibroblast response to the materials surface via regulating cell initial adhesion [47,48]. In this study, compared to the CAST group, the higher expression of VEGF and Col-I on SLM group might be the key point on promoting the adhesion of L-929 cells, which are consistent with the observation of SEM. Previous studies also found that adhesion-related gene expression depended on the concentration of Co-Cr ions [49]. Overall, compared with the CAST group, L-929 cells on SLM samples exhibit an earlier and stronger adhesion tendency on both the cytological and genetic levels.

The findings of this in vitro study suggest that the wear resistance and biocompatibility of Co-Cr alloys are related to microstructure and mechanical properties, which can be altered via manufacturing techniques. In addition, the SLM technique is considered a promising alternative for manufacturing dental alloys. Thus, the two null hypotheses were rejected.

There are several limitations in our study. First, compared with the L-929, human-derived cells may be able to better reflect the body condition. Second, the sample morphology is different from the dental restorations, and the results of sample characterizations only showed the inherent properties of the alloy. Finally, introducing the SLM technique to the dental field presents a wide spectrum of parameters that should be optimized, and more clinical tests must be investigated before a full recommendation for dental restorations.

## 5. Conclusions

This study presents important insights on the differences of wear resistance and biocompatibility of Co-Cr dental alloys fabricated via SLM and CAST techniques. Within the limitations of this study, the following conclusions can be derived:

- The friction coefficient curve of the SLM alloys was significantly stable and wear scars was shallower, compared with those of the CAST alloys. The stronger friction resistance of SLM alloys may be caused by the higher hardness.
- Based on the results of cell proliferation and cytotoxicity, the increasing cell number depended on time and the cell apoptosis rate of SLM alloy was significantly decreased, indicating a reduced deleterious effect on L-929 cells.
- The morphological observation and gene-expression results indicated that SLM alloys facilitate the initial L-929 cells’ spread and adherence, which were important criteria for biocompatibility evaluation.

Overall, the SLM technique can satisfy the demands of wear resistance and biocompatibility for Co-Cr alloy restorations. However, further animal experiments and clinical data are still needed to verify the results.

## Figures and Tables

**Table 1:** Primers’ sequences used in this study.

Target Gene	Forward Primer	Reverse Primer
GAPDH	AGGAGCGAGACCCCACTAACA	AGGGGGGCTAAGCAGTTGGT
VEGF	AGGAGTACCCCGACGAGATAGA	CACATCTGCTGTGCTGTAGGAA
Col-1	CACGGCTGTGTGCGATGA	TCGCCCTCCCGTCTTTG

## Data Availability

Not applicable.
